# How to define modeling languages?

**DOI:** 10.1007/s10270-023-01098-1

**Published:** 2023-03-20

**Authors:** Benoit Combemale, Jeff Gray, Bernhard Rumpe

**Affiliations:** 1 University of Rennes, Rennes, France; 2 University of Alabama, Tuscaloosa, AL USA; 3 RWTH Aachen University, Aachen, Germany

At the end of September 2022 at RWTH Aachen, Germany, there was a meeting attended by several experts on software language development with a specific emphasis on modeling. The LangDev meetings are particularly dedicated to the exchange of new ideas and innovations around the definition and use of Domain-Specific Languages (DSLs). Developers and users from multiple research and tooling groups attended, including representation from Essential, Freon (formerly ProjectIT), Gemoc, Langium, LinGo, MontiCore, MPS, Rascal, SpooFax, StarLasu, and Stratego. Attendees presented and discussed new trends and developments in their domains. Several practitioners also demonstrated new applications and uses of DSLs in various application domains, ranging from the European Union Digital COVID(-19) Certificate (the EU Digital Covid Certificate), to industrial printing systems, and digital twins, to name a few. LangDev 22 presented a very good balance among practitioners and researchers, as well as industrial and academic partners. The meeting welcomed a large number of new and young participants, which is a good sign for the community. Industrial partners included language workbench providers, and language workbench users, with impressive applications ranging from the EU DCC to examples in scientific computing. As the theme for this editorial, we want to share our impressions from that meeting as well as some general observations.

The diversity of language definition approaches appear to be more diverse, yet harmonize in the concepts available. Several techniques created originally in one technology stack are now adopted in other tooling platforms. It is also evident that various approaches are moving to the web, which allows developers to define new modeling languages without any need for local installation. However, there are also concerns that such an approach is nice to try out as a new technology, but when real intellectual property (IP) is involved, industrial companies will potentially prefer local, internet-free handling of their IP in the form of models. For such a need, company-internal clouds could be a solid alternative. What is really promising is that a number of software technologies are moving from configuration files at the very low-level (e.g., error prone JSON and Yaml) to well-defined DSLs.

Another intensive discussion focused on the development process for new languages. It is evident that defining very similar DSLs from scratch over and over again is inefficient for long-term sustainability. As an option, various forms of reuse were discussed. One type of reuse is a simple copy-paste-modify reuse, where DSL (n + 1) emerges as a specialized extension of its original DSL (n). Much better types of reuse exist in terms of black-box reuse, composition of DSLs from independently developed language components, and the need for reusable, standardized language component libraries that have been proposed and partially shown in successful application.

Model typing is connected to black-box reuse of language components and was raised as an important topic at LangDev 22. The issues include the transfer of traditional types known from programming languages to model elements describing classes, variables, objects, and expressions. Additionally, the notion of typing has to be transferred successfully to all other kinds of symbols that are present in models, such as pins, ports, channels, system components, actions, activities, and whatever concepts have been defined in the DSL. Finally, it was debated on whether the model itself should get a form of “type” in order to explain how the model can be connected to other models, leading to a well-typed composition technique, ideally with encapsulation, at the modeling level.

In addition to the above mentioned language composition and typing topics, LangDev 22 attendees also discussed projectional editing, multilevel modeling, parsing, compilers, transpilers, interpreters, as well as tool support (debugger, linter) for new DSL interfaces. Interestingly, simulation was not such a relevant topic, although the simulation community currently recognizes that the readability of simulation models is relevant for understandability and explanation of the simulation results. There also needs to be an efficient evolution of the simulation to explicit models, such as UML, SysML and especially simulation capable DSLs.

As a final note, Model-Driven Engineering has experienced success and is addressing more scenarios (e.g., digital twins, analytics) in an increasing number of application domains (e.g., natural systems, multi-physics systems). This broader use of MDE challenges the seminal, but yet limited, foundations on which most of the tools and methods are based, such as conformity and instantiation. New concerns, such as (multi-)fidelity, abstraction, precision, uncertainty, substitutability, accuracy, and validity are emerging very quickly, and need to be defined precisely. These concepts must be integrated into the core modeling techniques for DSLs to enable the sound development of the future generation of MDE approaches.

## In memory of Heinrich Hussmann (inaugural SoSyM editor)

This issue is a memorial to Heinrich Hussmann, our long-time friend and SoSyM editor. Heinrich was also the Diploma thesis advisor and later colleague of Bernhard. We are so sad that he suddenly passed away last year and have asked his closest colleagues, Manfred Broy, Albrecht Schmidt, and Martin Wirsing for the dedication (included in this issue) to his career and life.

Please find below a picture of SoSyM’s inaugural Editorial Board Meeting from October 2002 in Dresden during the 5th International Conference “UML 2002—The Unified Modeling Language: Model Engineering, Concepts, and Tools” (which was later renamed to MODELS). Heinrich organized that conference and he did a great job, even though there was a devastating flood a few months earlier (the web-servers had to be rescued after being offline and the conference venue also had to be changed). The first issue of SoSyM had just been released for this conference. Thus, Dresden was the birthplace of SoSyM.
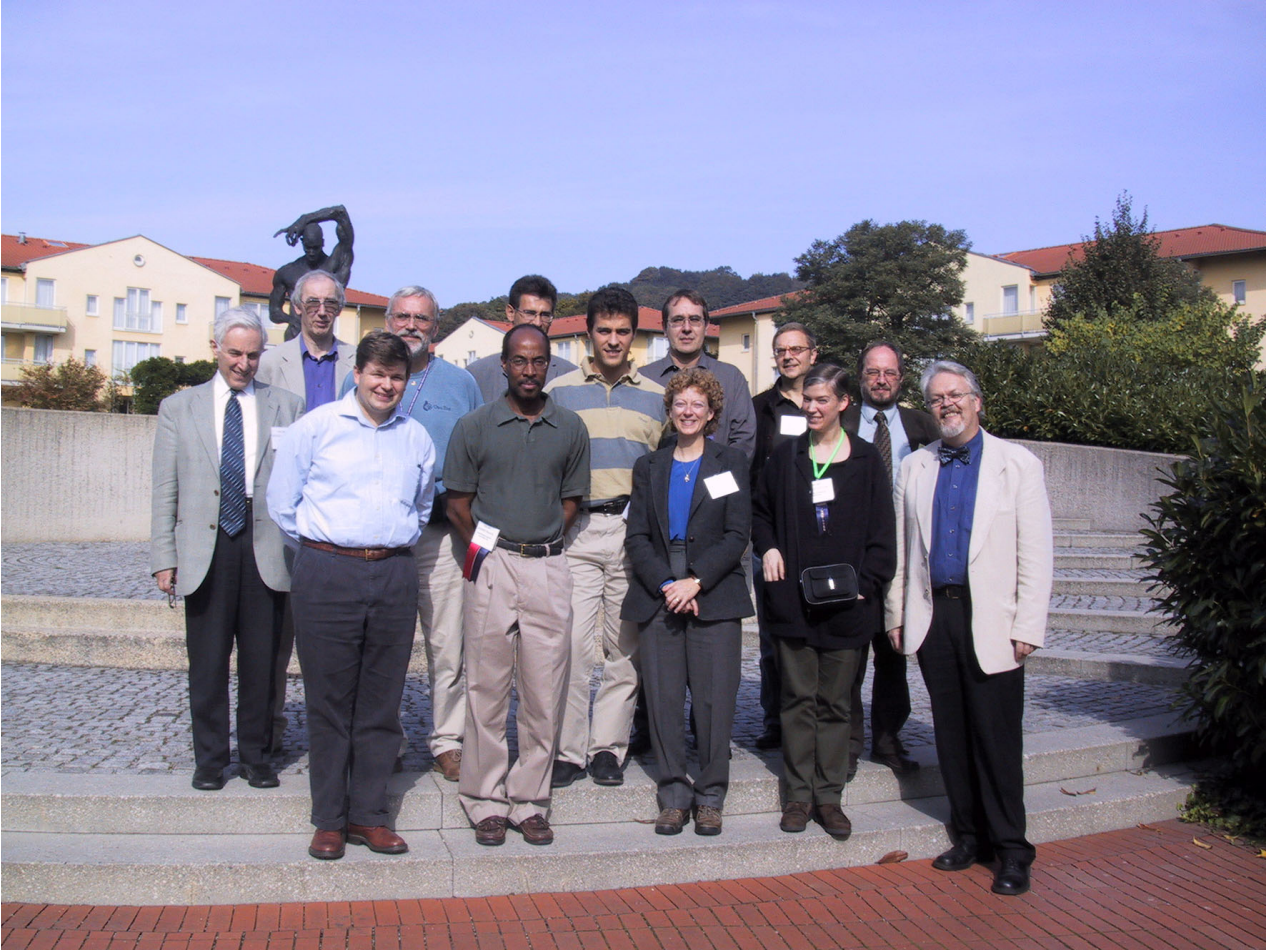


Inaugural SoSyM Meeting: October 2002 in Dresden, Germany (Heinrich in second row, far-right)

Heinrich, we miss you.

## Content of this issue

1. **Dedication to the Career and Life of Heinrich Hussmann**
  by Manfred Broy, Albrecht Schmidt, and Martin Wirsing.
2. **Expert Voice**
  “Trustworthy agent-based simulation: the case for domain-specific modelling languages” by Steffen Zschaler and Fiona A. C. Polack.
3. **FACS2021 Special Section**
  Guest editor: Gwen Salaün
4. **PoEM 2021 Special Section**
  Guest editors: Estefanía Serral, Janis Stirna, Jolita Ralyté, and Janis Grabis
5. **Regular Papers**
  “Dash: declarative behavioural modelling in Alloy with control state hierarchy” by Jose Serna, Nancy Day, and Shahram Esmaeilsabzali
  “Semi-automatic service value network modeling approach based on external public data” by Jingying Wang, Chao Ma, Huixin Xu, Zhiying Tu, Xiaofei Xu, Hanchuan Xu, and Zhongjie Wang

